# Antioxidant, Anti-Inflammation and Antiaging Activities of *Artocarpus altilis* Methanolic Extract on Urban Particulate Matter-Induced HaCaT Keratinocytes Damage

**DOI:** 10.3390/antiox11112304

**Published:** 2022-11-21

**Authors:** Chun-Yin Yang, Cheng-Chang Pan, Chih-Hua Tseng, Feng-Lin Yen

**Affiliations:** 1 School of Pharmacy, College of Pharmacy, Kaohsiung Medical University, Kaohsiung 807, Taiwan; 2 Sunray-Bio Biotech Co., Ltd., Kaohsiung 807, Taiwan; 3 Department of Fragrance and Cosmetic Science, College of Pharmacy, Kaohsiung Medical University, Kaohsiung 807, Taiwan; 4 Department of Medical Research, Kaohsiung Medical University Hospital, Kaohsiung 807, Taiwan; 5 Department of Pharmacy, Kaohsiung Municipal Ta-Tung Hospital, Kaohsiung 801, Taiwan; 6 Institute of Biomedical Sciences, National Sun Yat-Sen University, Kaohsiung 804, Taiwan; 7 College of Professional Studies, National Pingtung University of Science and Technology, Pingtung County 900, Taiwan; 8 Drug Development and Value Creation Research Center, Kaohsiung Medical University, Kaohsiung 807, Taiwan

**Keywords:** particulate matter, anti-pollution, *Artocarpus altilis*, keratinocytes, skin barrier

## Abstract

Particulate matter (PM) is one of the reasons that exacerbate skin diseases. Impaired barrier function is a common symptom in skin diseases, including atopic dermatitis, eczema and psoriasis. Herbal extracts rich in antioxidants are thought to provide excellent pharmacological activities; however, the anti-pollution activity of *Artocarpus altilis* extract (AAM) has not been investigated yet. The present study demonstrated that 5 μg/mL of AAM was considered to be a safe dose for further experiments without cytotoxicity. Next, we evaluated the anti-pollution activity of AAM through the PM-induced keratinocytes damage cell model. The results showed that AAM could reduce PM-induced overproduction of intracellular ROS and the final product of lipid peroxidation, 4-hydroxynonenal (4HNE). In addition, AAM not only reduced the inflammatory protein expressions, including tumor necrosis factor α (TNFα), TNF receptor 1 (TNFR1) and cyclooxygenase-2 (COX-2), but also balanced the aging protein ratio of matrix metalloproteinase (MMPs) and tissue inhibitors of metalloproteases (TIMPs) through downregulating the phosphorylation of mitogen-activated protein kinase (MAPK) signaling. For skin barrier protection, AAM could repair PM-induced barrier function proteins damage, including filaggrin, loricrin and aquaporin 3 for providing anti-aging bioactivity. In conclusion, AAM has the potential to be developed as an anti-pollution active ingredient for topical skin products to prevent skin oxidation, inflammation and aging, and restore the skin barrier function.

## 1. Introduction

Air pollution is a health hazard problem of great concern to the public; in particular, PM is the most important indicator of air pollution. According to the Health Effects Institute (HEI), air pollution has been highly associated with approximately 5 million deaths globally and became the fifth leading risk factor for mortality worldwide in 2017 [1]. Particulate matter (PM) produced by transports, power generation, dust, biological decay, agricultural waste and industry are believed to be a long-term hazard to our respiratory system, cardiovascular system [2] and skin barrier function [3]. It is a complex mixture of solid and liquid particles including ionic substances, metals, organic carbons, polycyclic aromatic hydrocarbons (PAHs) and other unidentified constituents [4]. PM has been classified as carcinogenic to human beings by WHO’s specialized cancer agency, the International Agency for Research on Cancer (IARC) [5]. Previous research had pointed out that overexposure to PM may lead to poor wound healing, acne, psoriasis, atopic dermatitis, contact dermatitis and skin cancer [1]. Briefly, PM is responsible for a large amount of health-related problems worldwide.

Normal skin barrier function provided enough skin integrity and maintained skin hydration and defense against extrinsic substances, such as environmental allergens, UV rays and pollutants. Stratum corneum (SC) is the outermost layer of the skin, which may be disrupted by oxidation and prolonged inflammation [6]. A previous study indicated that chronic exposure to PM potentially triggered excessive oxidative stress and inflammation behavior [7,8,9]. These processes are almost interlinked and both are strongly related to the overproduction of reactive oxygen species (ROS). Filaggrin (FLG), involucrin (INV) and loricrin (LOR) are the major epidermal barrier proteins that affect keratinocyte differentiation and maintain the physical strength of the skin [10]. In particular, filaggrin plays a pivotal role in the regulation of skin hydration, pH adjustment and photoprotection. Early research demonstrated that PM degraded barrier proteins by inducing an inflammatory response, such as the expression of cyclooxygenase-2 (COX-2) and the activation of tumor necrosis factor α receptor (TNFR) by tumor necrosis factor α (TNFα), and, finally, lead to skin dysfunction [3,10,11]. Abnormal SC leads to decreased skin defenses ability, increased transepidermal water loss (TEWL) and the progressive loss of skin integrity. Skin dysfunction due to PM exposure contributed to the promotion and exacerbation of several skin diseases, for instance, erythema, edema, premature skin aging, sensitization, atopic dermatitis, contact dermatitis and psoriasis [1,12].

*Artocarpus altilis* (*A. altilis*), a high-economic-value evergreen tree that widely grows in subtropical and tropical areas, is used in traditional medicine for treating infection, inflammation and diarrhea commonly. *A. altilis* methanolic extract (AAM) exerts potent skin-whitening effects through the activation of MAPK signaling pathway to degrade the microphthalmia-associated transcription factor (MITF) then decrease synthesis of tyrosinase and melanin production [13]. In addition, AAM provided a great UV protection effect through the decreasing of ROS generation and lipid peroxidation to reduce the secretion of TNF-α and interleukin-1 beta (IL-1β), and finally, inhibit the expression of cytosolic phospholipase A2 (cPLA2), COX2, vascular cell adhesion protein 1 (VCAM-1) [14]. The main compound in AAM is artocarpin, a prenylated isoflavone with various bioactivities. Both in vitro and in vivo studies have suggested that artocarpin showed protection against UV radiation [15,16], cancers [17,18,19] and an accelerated wound healing rate [20]. However, the anti-pollutant bioactivity of AAM has not been investigated yet, we predicted that AAM containing rich artocarpin would give beneficial effects on skin damage stimulated by PM.

The aim of the present study was to determine the potential mechanism of antioxidant and anti-inflammatory bioactivities of AAM on PM-stimulated HaCaT keratinocytes through the MAPK signal transduction pathway that is involved.

## 2. Materials and Methods

### 2.1. Extraction Methods of Artocarpin altilis

The heartwood of *A. altilis* was obtained from the Tainan District Agricultural Research and Extension Station (Tainan DARES, Tainan, Taiwan). The plant species was identified by Dr. Ming-Hong Yen of the Graduate Institute of Natural Products, College of Pharmacy, Kaohsiung Medical University, Kaohsiung, Taiwan. The extraction methods of AAM were described previously [21]. Briefly, a total of 200 g of dried, chipped heartwood pieces of *A. altilis* were mixed with 4 L of methanol (Aencore Chemical, Surrey Hills, Australia) and extracted by an ultrasonic bath (Branson 5510, Emerson Electric, Brookfield, CT, USA) for 1 h. The process was repeated twice, and the liquid extract was filtered, concentrated and then lyophilized to obtain AAM. The extraction yield of AAM was calculated by the equation below. For further experiments used, AAM was stored in a moisture-proof container at room temperature.

$$\mathrm{Yield}\;\left(\%\right)=\frac{\mathrm{AAM}\;\mathrm{extract}\;\mathrm{weight}\;(g)}{\mathrm{amount}\;\mathrm{of}\;\mathrm{dry}\;\mathrm{AA}\;\mathrm{heartwood}\;(g)}\times 100\%$$

### 2.2. Quantification of the Index Component of AAM

High-performance liquid chromatography (HPLC) was used to analyze the content of the index component, artocarpin, in each batch of AAM. The artocarpin standard curve was obtained by the HPLC analysis system (LaChrom Elite L-2000, L-2130 pump, L-2200 autosampler and L-2420 UV–vis detector, Hitachi, Tokyo, Japan) with Mightysil RP-18 GP column (250 × 4.6 mm, i.d., 5 μm, Kanto Corporation, Portland, OR, USA). Isocratic elution was performed by using a mobile phase that contained methanol: deionized distilled water (9:1, *v*/*v*) for 8 min. The samples were then eluted at a flow rate of 1 mL/min at 282 nm. The injection volumes of all samples and standard solutions were 20 μL. The artocarpin standard curve showed good linearity (r^2^ > 0.999) at the concentration range from 0.05 to 100 μg/mL.

### 2.3. Cell Line and Culture

The human skin keratinocytes (HaCaT cells) cell line was obtained by Istituto Zooprofilattico Sperimentale della Lombardia e dell’Emilia Romagna (Brescia, Italy). HaCaT cells were cultured in Dulbecco’s modified Eagle’s Medium (DMEM) supplemented with 10% fetal bovine serum (FBS) and 1% penicillin-streptomycin-amphotericin B (PSA; New Haven, CT, USA). The HaCaT cells were grown in a 37 °C incubator with 5% CO_2_. The medium was changed every 2 days, and passages from 5 to 15 were used for experiments.

### 2.4. Preparation of Particulate Matter

The standard urban dust PM (SRM 1649b) was purchased from the National Institute of Standards and Technology (NIST, Gaithersburg, MD, USA). PM particles were suspended in PBS and sonicated for 10 min in an ultrasonic bath to avoid particle aggregation. The stock PM suspension was prepared to obtain a concentration of PM at 10 mg/mL in PBS.

### 2.5. Cell Viability Assay

Cell viability was evaluated using 3-(4,5-dimethylthiazol-2-yl)-5-(3-carboxymethoxyphenyl)-2-(4-sulfophenyl)-2H-tetrazolium (MTS) reagent (BioVision, Waltham, MA, USA). HaCaT cells were seeded into 96-well microplates (1.2 × 10^5^ cells/well), and the attachment of adherents was allowed and incubated at 37 °C for 18–24 h. The HaCaT cells were treated with different concentrations of AAM (dissolved in DMSO) in the medium without FBS for 24 h. Then, 20 μL of MTS solution was then added and incubated for 1.5 h at 37 °C. The optical absorption at 490 nm was determined using a microplate spectrophotometer (SpectraMax^®^ ABS Plus, Molecular Devices, San Jose, CA, USA).

### 2.6. Reactive Oxygen Species (ROS) Assay 

The cellular ROS generation was measured using DCFDA/H2DCFDA Assay. HaCaT cells were seeded into 96-well microplates (1 × 10^5^ cells/well), the attachment of adherents was allowed and incubated at 37 °C for 18–24 h. Subsequently, cells were pre-treated with AAM for 3 h follow-up staining with 100 μL of 20 μM dichlorodihydrofluorescein diacetate (DCFH-DA; Sigma, St. Louis, MO, USA). After 30 min, cells were incubated with 50 μg/cm^2^ PM suspension for one hour then detected relative fluorescence intensity by fluorescent spectroscopy (BioTek, Winooski, VT, USA) with excitation/emission wavelength at 485 nm/535 nm. The relative ROS generation was calculated using the equation below:

$$\mathrm{Relative}\;\mathrm{ROS}\;\mathrm{generation}=\frac{\mathrm{Fluorescence}\;\mathrm{intensity}\;\mathrm{of}\;\mathrm{sample}}{\mathrm{Fluorescence}\;\mathrm{intensity}\;\mathrm{ofcontrol}}$$

### 2.7. Western Blot Analysis

The PM-induced HaCaT cells model was modified by Huang et al. [22]. HaCaT cells were seeded into 6-well plates (4 × 10^5^ cells/well). Subsequently, cells were pre-treated with AAM 2.5 and 5 μg/mL for 3 h. After that, treated with PM (50 μg/cm^2^) suspension for various timepoints. Then, the cells were washed twice with PBS, lysed with a RIPA lysis buffer (0.5 M Tris-HCl, pH 7.4, 1.5 M NaCl, 2.5% deoxycholic acid, 10% NP-40, 10 mM EDTA) containing protease and phosphatase inhibitors. The collected samples were quantified the total protein content with the bicinchoninic acid protein assay kit (BCA kit; Thermo Fisher Scientific, Waltham, MA, USA). A total of 20 μg of total protein were separated using 10% sodium dodecyl sulfate-polyacrylamide gel electrophoresis (SDS-PAGE). Following this, the proteins were transferred onto PVDF membranes (Merck Millipore). After blocking for 1 h, the membranes were incubated overnight with primary antibodies, including AQP3, TNF-α (1:1000 dilution, ABclonal, Woburn, MA, USA), COX-2, MMP-2, TIMP1, phospho-SAPK/JNK (1:1000 dilution, Cell Signaling Technology, Danvers, MA, USA), 4HNE, p-p38α (1:1000, Merck Millipore Corporation, Billerica, MA, USA), p-ERK (1:2500, Merck Millipore Corporation, Billerica, MA, USA), Loricrin, MMP-1 (1:1000, Proteintech, Chicago, IL, USA), Involucrin (1:1000, Arigo Biolaboratories, Hsinchu, Taiwan, China), filaggrin (1:500, Santa Cruz Biotechnology, Dallas, TX, USA) and GAPDH (1:2500, Santa Cruz Biotechnology, Dallas, TX, USA) at 4 °C. After washing with Tris-buffered saline with 0.05% Tween-20 (TTBS), membranes were incubated with secondary antibodies for 1 h at room temperature, and then, washed three times with TTBS again. The immunoreactive bands of each sample were reacted with enhanced chemiluminescence reagents and visualized using the Touch Imager (e-BLOT; Shanghai, China). Antibodies against GAPDH were used as the internal control.

### 2.8. Statistical Analysis

All the data were performed in triplicate and were represented as mean ± standard deviation (SD) using Microsoft Excel 2019 software (Microsoft Office, Microsoft Corporation, Redmond, WA, USA). Statistical significance between multiple groups was analyzed by one-way ANOVA with Tukey’s post hoc test using SPSS 20 software (SPSS Inc., Chicago, IL, USA). *p* value < 0.05 was considered as the statistically significant level.

## 3. Results and discussion

### 3.1. The Yield and Artocarpin Content of AAM

**Figure 1:**
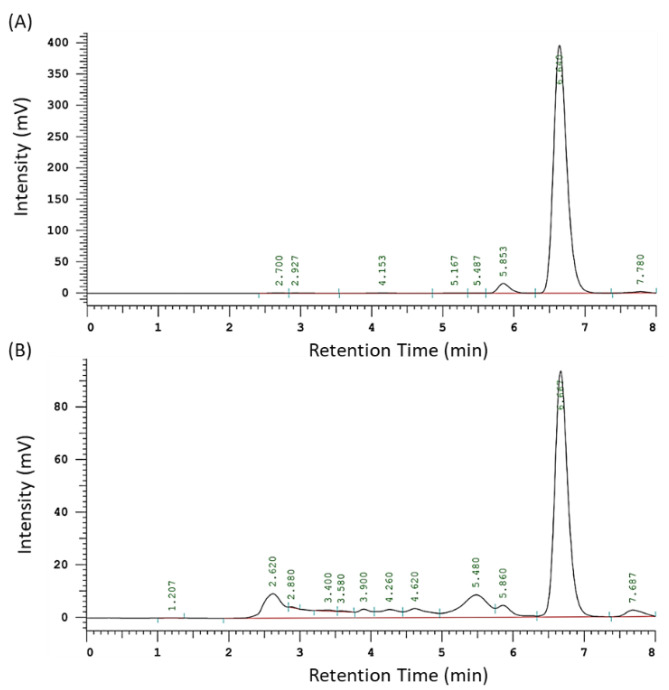
HPLC chromatography of (**A**) artocarpin at 100 µg/mL and (**B**) AAM at 100 µg/mL.

The yield of the AAM extract was approximately 3.1%. The retention time of artocarpin was 6.6 min in HPLC chromatogram (Figure 1A). Moreover, AAM chromatography also provided an obviously high peak at 6.6 min (Figure 1B), which could be identified as the index component, artocarpin. The quantification result of the artocarpin content of AAM, in this study, was about 225 µg artocarpin per mg AAM.

### 3.2. Effects of AAM on HaCaT Keratinocytes Cell Viability

**Figure 2:**
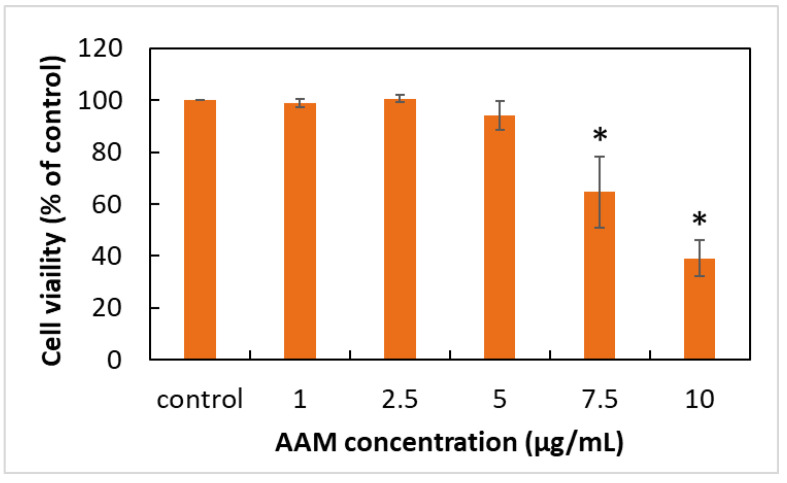
The effects of AAM on HaCaT keratinocytes cell viability. Data were from three independent experiments. Value are mean ± SD (*n* = 3). * Significantly different from control group (*p* < 0.05).

In recent years, several studies revealed that supplying enough anti-oxidants had been found to attenuate oxidative damage, inflammation and apoptosis on skin cells, such as resveratrol [7], eckol [23], glycofullerens [24] or herbal extracts including Astragali Radix [9], *Opuntia Humifusa* [25] or *Cornus Officinalis* [26]. However, the mechanism of AAM to protect the skin against PM-induced skin damage has not been investigated yet. Thus, we first evaluated the safety dose of AAM, cell viability of AAM with 1% DMSO treatment in HaCaT cells was determined using the MTS assay as shown in Figure 2. No cell lysis or discrete intra-cytoplasmatic granules were observed at concentrations less than 5 μg/mL when compared to the control group containing DMEM with 1% DMSO. This finding demonstrated that AAM less than 5 μg/mL did not display any obvious cell toxicity. The further study decided to use AAM at concentrations of 2.5 and 5 μg/mL to evaluate the biological activity of AAM in PM-induced keratinocytes damage.

### 3.3. AAM Inhibited PM-Stimulated Oxidative Stress on HaCaT Cells

**Figure 3:**
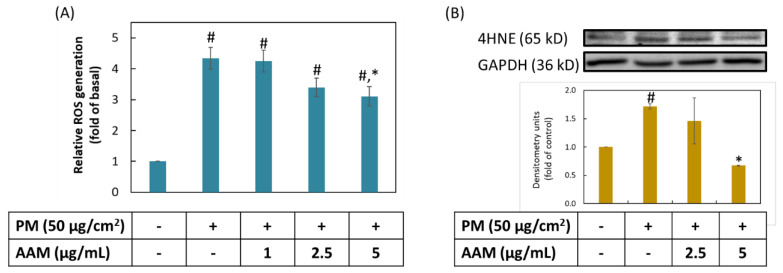
The antioxidant activity of AAM on PM-induced ROS production (**A**) and 4HNE lipid peroxidation ending product (**B**) in HaCaT keratinocytes. Data are shown as the mean ± SD of three independent experiments; # Significantly different from control group (*p* < 0.05); * Significantly different from PM treatment alone (*p* < 0.05).

ROS was regarded as a group of active oxidative substances, which involved in several pathological phenomenon such as cardiovascular disease, diabetes and cancer. Oxidative stress refers to the increasing level of intracellular ROS and has been shown to damage protein, lipid and nucleic acid [27]. The previous review also pointed out that the promotion of the oxidation of ω-6 polyunsaturated fatty acids was closely related to the increasing levels of ROS [28]. The surface of PM contained heavy metal ions which initiated lipid peroxidation when contact with cells [29]. 4-hydroxynonenal (4HNE) was an ending product of ROS-mediated lipid peroxidation, in response to oxidative stress and damaged cells and tissues [30]. As shown in Figure 3, HaCaT cells significantly increased ROS production and 4HNE protein expression after treatment with PM when compared to the control group (*p* < 0.05). Pretreatment with AAM at 5 μg/mL not only reduce PM-induced overproduction of the ROS, but also decreased the protein expression of 4HNE (*p* < 0.05). These findings demonstrated that AAM at 5 μg/mL is a good antioxidant to prevent PM-induced oxidative stress in HaCaT cells.

### 3.4. Inhibition of AAM on PM-Induced Inflammation in HaCaT Cells

**Figure 4:**
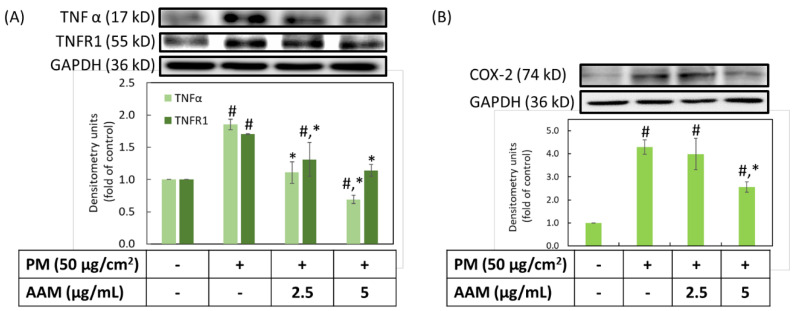
Effect of AAM on PM-induced inflammatory protein expressions in HaCaT cells, TNFα and TNFR1 (**A**) and COX-2 (**B**). Data were from three independent experiments. Value are mean ± SD (*n* = 3). # Significantly different from control group (*p* < 0.05); * Significantly different from PM treatment alone (*p* < 0.05).

A previous study provided evidence that TNFα played a crucial role in an early event on PM-inhibited skin barrier proteins function; moreover, the blocking of TNFR1 could decrease the changes in barrier proteins [3,10]. Furthermore, COX-2 is a well-known inflammatory protein that could only be detected in cells under inflammatory stimuli, such as UVB and PM [31]. As shown in Figure 4, the expression of TNFα, TNFR1 and COX-2 were about 1.9, 1.7 and 4.3 times higher than the control group in HaCaT keratinocytes, respectively (*p* < 0.05). It is very exciting that AAM at concentrations of both 2.5 and 5 μg/mL could effectively inhibit inflammatory protein expressions, such as TNFα and TNFR1. However, only 5 μg/mL could suppress COX-2 expression when compared to the PM-induced group (*p* < 0.05). These findings demonstrated that AAM at 5 μg/mL is a good anti-inflammatory ingredient to prevent PM-induced inflammation in HaCaT cells. Cho YJ et al. showed a similar result that curcumin ameliorated radiation-induced lung inflammation and fibrosis through suppression of TNFα and TNFR1. Additionally, curcumin inhibited the COX-2 expression via the regulation of NF-κB levels [32].

### 3.5. AAM Suppressed PM-Induced Phosphorylation of MAPK Signaling Pathway

**Figure 5:**
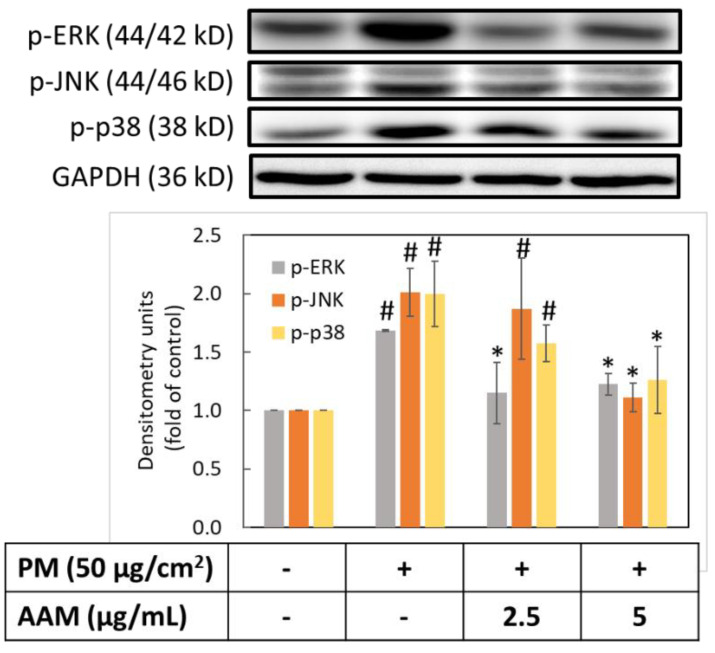
Effect of AAM on PM-induced phosphorylation of MAPK proteins in HaCaT cells. Data were from three independent experiments. Value are mean ± SD (*n* = 3). # Significantly different from control group (*p* < 0.05); * Significantly different from PM-induced group (*p* < 0.05).

Since ROS activated phosphorylation of MAPK and NF-κB signaling pathways to increase the amount of pro-inflammatory cytokines. Therefore, we evaluated the phosphorylation of ERK, JNK and p38. Figure 5 showed that PM could increase the phosphorylation of p-ERK, p-JNK and p-p38 when compared to the control group (*p* < 0.05). In contrast, when 5 μg/mL of AAM was pretreated, the protein expressions of MAPK phosphorylation in PM-induced HaCaT cells were significantly inhibited (*p* < 0.05). Phosphorylation of MAPK is involved in skin inflammation and aging process through mediated the expression of COX-2 and MMPs protein expression [33]. AAM possessed anti-aging and anti-inflammatory effects by regulating the phosphorylation of MAPK-signaling proteins.

### 3.6. AAM Suppressed PM-Induced Aging-Related Protein Expression

**Figure 6:**
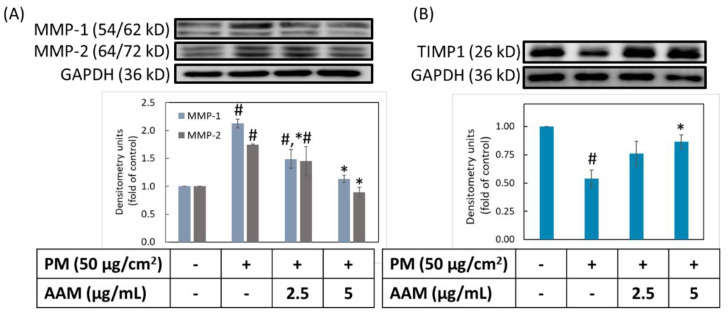
Effect of AAM on PM-induced aging protein expressions in HaCaT cells. MMP-1 and MMP-2 (**A**), TIMP1 (**B**). Data were from three independent experiments. Value are mean ± SD (*n* = 3). # Significantly different from control group (*p* < 0.05); * Significantly different from PM treatment alone (*p* < 0.05).

Matrix metalloproteinase (MMPs) are extracellular proteinases that degrade various extracellular matrices (ECM) in the skin, such as different types of collagen and elastin [34]. Degradation of ECM led to wrinkle formation and skin sagging. In addition, tissue inhibitors of metalloproteases (TIMPs) played regulatory roles to inhibit the expression of MMPs for preventing skin aging. There are several factors, such as UV overexposure and smoke that easily disturbed the balance between TIMPs/MMPs to degrade the ECM, and resulted in skin sagging and wrinkles. In our study, the level of MMP-1 and MMP-2 were obviously increased after stimulated with PM, but the level of TIMP1 was decreased (Figure 6). Pretreatment with 2.5 and 5 μg/mL of AAM showed a dose-dependent inhibited MMP-1 expression but for MMP-2 only 5 μg/mL of AAM could significantly decrease the expression level (*p* < 0.05). Moreover, 5 μg/mL of AAM also simultaneously increased TIMP1 expression. These findings demonstrated that AAM at 5 μg/mL is a good antiaging ingredient to prevent PM-induced skin aging in HaCaT cells. Kwon et al. also reported similar results that *Spatholobus Suberectus* stem extract (SS) attenuated UVB-induced photoaging via modulation of MAPK and MMPs signaling. SS inhibited MMP-1 expression, upregulated TIMP1 and blocked UVB-induced phosphorylation of MAPKs in human keratinocytes [35].

### 3.7. AAM Prevented PM-Induced Skin Barrier Dysfunction

**Figure 7:**
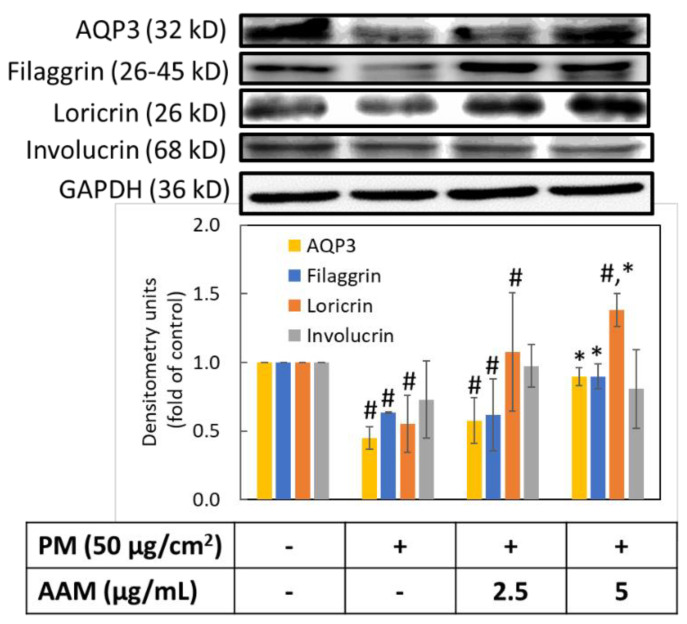
Effect of AAM on PM-induced skin barrier dysfunction in HaCaT cells. Data were from three independent experiments. Value are mean ± SD (*n* = 3). # Significantly different from control group (*p* < 0.05); * Significantly different from PM treatment alone (*p* < 0.05).

Skin barrier proteins are closely related to the barrier function, including skin integrity, mechanical resistance, stability and transepidermal water loss (TEWL) [3]. Skin barrier dysfunction and impairment of skin hydration have been considered the major reasons for the pathogenesis of various skin problems such as atopic dermatitis, eczema, psoriasis, premature aging and delayed wound healing [6,36,37]. Cells exposed to PM produce a large amount of TNF-α and triggered the release of cytokines due to TNF-α binding to TNFR1 [38]. The activation of TNFR1 leads to the generation of a series of inflammatory responses. Thus, we further evaluated the water channel protein aquaporin 3 (AQP3) and the barrier structure proteins, including filaggrin (FLG), loricrin (LOR) and involucrin (INV). Figure 7 revealed that the PM group significantly decreased the protein expressions of AQP3, FLG and LOR in HaCaT cells (*p* < 0.05), but there was no significant difference in INV expression. Similarly, Kim et al. determined that TNF-α also related to the regulation of barrier proteins (FLG, LOR, INV). TNF-α reduced the expression level of FLG and LOR, which increased TEWL and reduced skin healing ability and moisturizing ability [10]. Whereas, AAM pretreatment at the concentration of 5 μg/mL could effectively increase the expression of AQP3, FLG and LOR (*p* < 0.05), but still has no effect on involucrin. These findings indicated that AAM at 5 μg/mL is a good moisturizer to prevent PM-induced skin barrier dysfunction in HaCaT cells.

## 4. Conclusions

**Figure 8:**
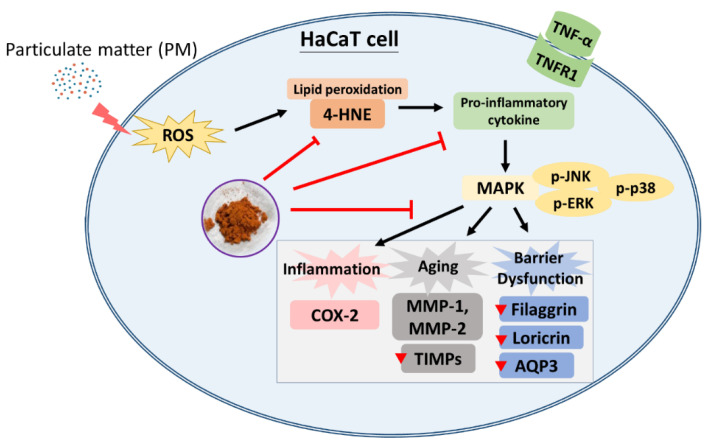
A proposed skin protection mechanism of AAM on PM-induced HaCaT keratinocytes.

The possible mechanisms of AAM skin protection bioactivities against PM are summarized in Figure 8. Our study revealed that AAM protects skin from PM-damage through the regulation of the MAPK signaling pathway to reduce oxidative stress, inflammation, aging and barrier dysfunction. Therefore, we consider that AAM would be a potential candidate for improving skin barrier integrity and can be developed as a useful active ingredient in the cosmetics industry. Last but not least, there are still some limitations to this study. It is worth looking forward to the formulation design and the clinical trial in the future to evaluate the efficiency and side effects of AAM.

## Data Availability

All data presented in the study are available on request from the corresponding author (flyen@kmu.edu.tw).
